# Non‐native ants drive dramatic declines in animal community diversity: A meta‐analysis

**DOI:** 10.1111/icad.12672

**Published:** 2023-07-29

**Authors:** Maximillian P. T. G. Tercel, Jordan P. Cuff, William O. C. Symondson, Ian P. Vaughan

**Affiliations:** ^1^ School of Biosciences Cardiff University Cardiff UK; ^2^ Durrell Wildlife Conservation Trust Les Augrès Manor Jersey Channel Islands; ^3^ School of Natural and Environmental Sciences Newcastle University Newcastle upon Tyne UK

**Keywords:** biodiversity decline, biological invasion, community response, Formicidae, global change, insect declines, introduced species, invasional meltdown, native diversity

## Abstract

1. Non‐native ants can cause ecosystem‐wide ecological change, and these changes are generally assumed to be negative. Despite this, the evidence base has never been holistically synthesised to quantify whether and to what degree non‐native ants impact native species diversity.
2. In this study, we performed a meta‐analysis of the effects of ant invasion on animal communities. We extracted data from 46 published articles investigating abundance (156 effect sizes) and richness (53 effect sizes) responses of animal taxa to ant invasion in locations relatively unimpacted by other stressors (e.g. human disturbance, other non‐native species) to help isolate the effects of invasion.
3. Overall, local animal diversity declined severely, with species abundance and richness lower by 42.79% and 53.56%, respectively, in areas with non‐native ants compared with intact uninvaded sites. We then combined responses of individual animal taxa extracted from an article into a single response to represent the ‘community’ abundance (40 effect sizes) or richness (28 effect sizes) response to non‐native ants represented in each article. Local communities decreased substantially in total abundance (52.67%) and species richness (53.47%) in invaded sites.
4. These results highlight non‐native ants as the drivers, rather than passengers, of large net‐negative reductions to animal community diversity in relatively undisturbed systems around the world, approximately halving local species abundance and richness in invaded areas. Improved international prevention processes, early detection systems harnessing emerging technologies, and well‐designed control measures deployable by conservation practitioners are urgently needed if these effects are to be mitigated, prevented or reversed.

## Graphical abstract

- Non‐native ants are assumed to reduce native animal community diversity, but potential impacts have never been holistically synthesised at the global scale.
- A meta‐analysis of 209 studies from 46 articles examining animal community richness and abundance in paired sites with and without invasive ants shows an approximate 50% reduction in community diversity.
- These impacts are found across five continents in both vertebrate and invertebrate taxa, highlighting invasive ants as the drivers, rather than passengers, of strong negative diversity declines of native species.

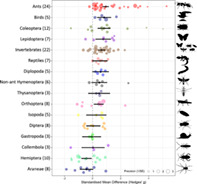

## INTRODUCTION

The diversity of life on Earth is integral to a healthy and stable environment, underpinning environmental resilience (Folke et al., 2004) and providing all organisms, including humans, with the life systems required to survive. Invasive species (organisms introduced outside of their natural range that negatively affect native species) are a threat to global biodiversity (Luque et al., 2014; Simberloff et al., 2013), often leading to the homogenisation of ecosystems (McKinney & Lockwood, 1999). In ‘100 of the World's Worst Invasive Alien Species’, the International Union for the Conservation of Nature (IUCN) lists five invasive ant species (Lowe et al., 2000; Luque et al., 2014). Ants are ecologically important social insects, participating in a wide range of species interactions, for example, as predators, parasites, herbivores, granivores, prey, mutualists and hosts, across almost all terrestrial environments and all continents except Antarctica (Hölldobler & Wilson, 1990; Lach et al., 2010; Parker & Kronauer, 2021; Stadler & Dixon, 2005). Invasive ants possess adaptations such as supercoloniality and dietary generalism to establish themselves outside of their natural ranges and subsequently ecologically dominate native communities (Baratelli et al., 2023; Holway et al., 2002; Wong et al., 2023). Studies investigating native species responses to ant invasion tend to show negative consequences, but many studies cannot isolate non‐native ants as the causal factor of these changes due to environmental differences between uninvaded and invaded sites or other confounding variables (Hill et al., 2003; King & Tschinkel, 2008; King & Tschinkel, 2013; Narendra et al., 2011; Sakamoto et al., 2019; Stuble et al., 2013; Vonshak et al., 2010). Typically, non‐native ants are found in heavily disturbed habitats because of their transportation by humans around the world (McGlynn, 1999; Suarez et al., 2010) and because they are thought to be disturbance specialists (Achury et al., 2021; Berman et al., 2013; Holway et al., 2002; Menke et al., 2018), thriving in structurally open and homogenous environments. Measuring local community responses to non‐native ants in these areas might therefore confuse results because of an already diminished native community and the presence of other non‐native species (Berman et al., 2013; Stuble et al., 2013).

Invasive ants are generally expected to lower native species diversity through direct predation and competition, as well as indirect effects arising from the extirpation of certain species. Such effects have been observed from studies examining native ant responses to invasive ants (Cooling & Hoffmann, 2015; Dunham & Mikheyev, 2010; Hoffmann et al., 1999; Hoffmann & Parr, 2008), but there are mixed responses from other taxa (Alvarez‐Blanco et al., 2017; Dunham & Mikheyev, 2010; Estany‐Tigerström et al., 2010; McPhee et al., 2012; Porter & Savignano, 1990). The fate of a given species is likely determined by the way in which it might interact with any incoming non‐native ants, if they interact at all. For example, native scale insects may benefit from highly aggressive non‐native ants that can protect them more effectively from natural enemies than a native ant. However, necessarily, the natural enemies of the scale insect and the native ants might be adversely affected in this scenario. Furthermore, local species might be indirectly affected by incoming non‐native ants at the community level. Studies describing ‘invasional meltdown’ support this idea, whereby invasive ants cause ecosystem‐wide devastation as a result of cascading direct and indirect species responses to invasion (Handler et al., 2007; O'Dowd et al., 2003; O'Loughlin & Green, 2015).

The current evidence base suggests the impact of non‐native ants can range from ecological damage at the ecosystem scale to being beneficial for some native taxa. This body of research has not yet been synthesised holistically in relatively intact natural systems. Robust estimates of local animal community responses to non‐native ants in primarily native undisturbed habitats would be a timely addition to the ecological knowledge base and could yield insights that can be used to inform biodiversity conservation.

Here, we conduct a quantitative assessment of local species responses to non‐native ants across many taxa and environments around the world using a meta‐analytical approach. We use the term ‘local’ instead of ‘native’ because, although studies were stringently selected solely in relatively undisturbed natural systems, we cannot rule out that a small percentage of species in these areas may be non‐native. We quantify the effects of non‐native ants on local animal abundance and richness at both the level of an individual taxon (e.g. Coleoptera, Lepidoptera, birds, and reptiles) and averaged across all taxa in a local community (the mean response of all taxa in a given article). We compute local responses by comparing abundance and species richness values in native habitats invaded by non‐native ants (but otherwise undisturbed) to paired uninvaded control sites with nearly identical environmental conditions. In doing so, we answer four key questions: (1) What is the overall impact of non‐native ants on local species abundance and richness around the world? (2) Are responses taxon‐specific? (3) Are responses dependent on the unique local community being invaded? and (4) To what extent are responses determined by non‐native ant species, habitat type or location of the study?

## METHODS

### Data collection

We aimed to compile a comprehensive database of articles reporting the effect of non‐native ants on local species richness and/or abundance that adhered to our criteria. These articles were identified using Web of Science as our search engine, using the Web of Science Core Collection, BIOSIS Citation Index, KCI‐Korean Journal Database, MEDLINE, Russian Science Citation Index and SciELO Citation Index databases for articles published between 1900 and 2023 using a Boolean search string (Appendix S1). This returned 800 articles on 30 January 2023. A PRISMA flow diagram (Figure S1) shows the stages at which articles were disqualified or eventually used in the current study.

To be suitable for our database, articles needed to adhere to the following criteria: (1) report the abundance and/or species (or morphospecies) richness of local species in paired uninvaded and invaded sites, before and after invasion by invasive ants or before and after eradication of invasive ants (for the latter two, multiple years of sampling and environmental variables were required to account for interannual differences in local community); (2) investigate community‐wide effects, not the response of a single species, unless recording the response of native vertebrates to invasion, which are typically single‐species studies; (3) undertake observations in relatively natural environments primarily made up of native vegetation; (4) sites were generated randomly within each treatment; (5) any changes to local species diversity were directly attributable to, or very likely to be caused by, non‐native ants (i.e. no other non‐native species were highly abundant); (6) report data with mean, sample size and variance (standard deviation, standard error or confidence intervals), or in another format that allowed these statistics to be inferred from the reported results, such as plots; and (7) published in English. Sample sizes for uninvaded and invaded groups were the number of distinct sites reported by the authors.

We extracted data for each local taxon response to non‐native ants from each article using a data extraction spreadsheet (Table S1), and hereafter refer to these as separate ‘studies’. These are observations of the species richness or abundance of a given taxon in geographically discrete paired sites, one with non‐native ants present (invaded) and the other with non‐native ants absent (uninvaded). Articles may report more than one study, for example, an article may report the species richness of beetles (one study) and the abundance of native lizards (a second study). From studies reporting local species diversity changes before and after non‐native ant eradication/control (richness = 5 of 53 studies, abundance = 19 of 156 studies), we used mean values for before and after years but, where possible, excluded diversity values from the first year after eradication to allow local communities to respond to the removal of introduced ants. To be considered ‘uninvaded’, non‐native ants had to be completely removed or in very low numbers (0–1 individual ants per uninvaded site, which were typically ≥20 m^2^).

**FIGURE 1:**
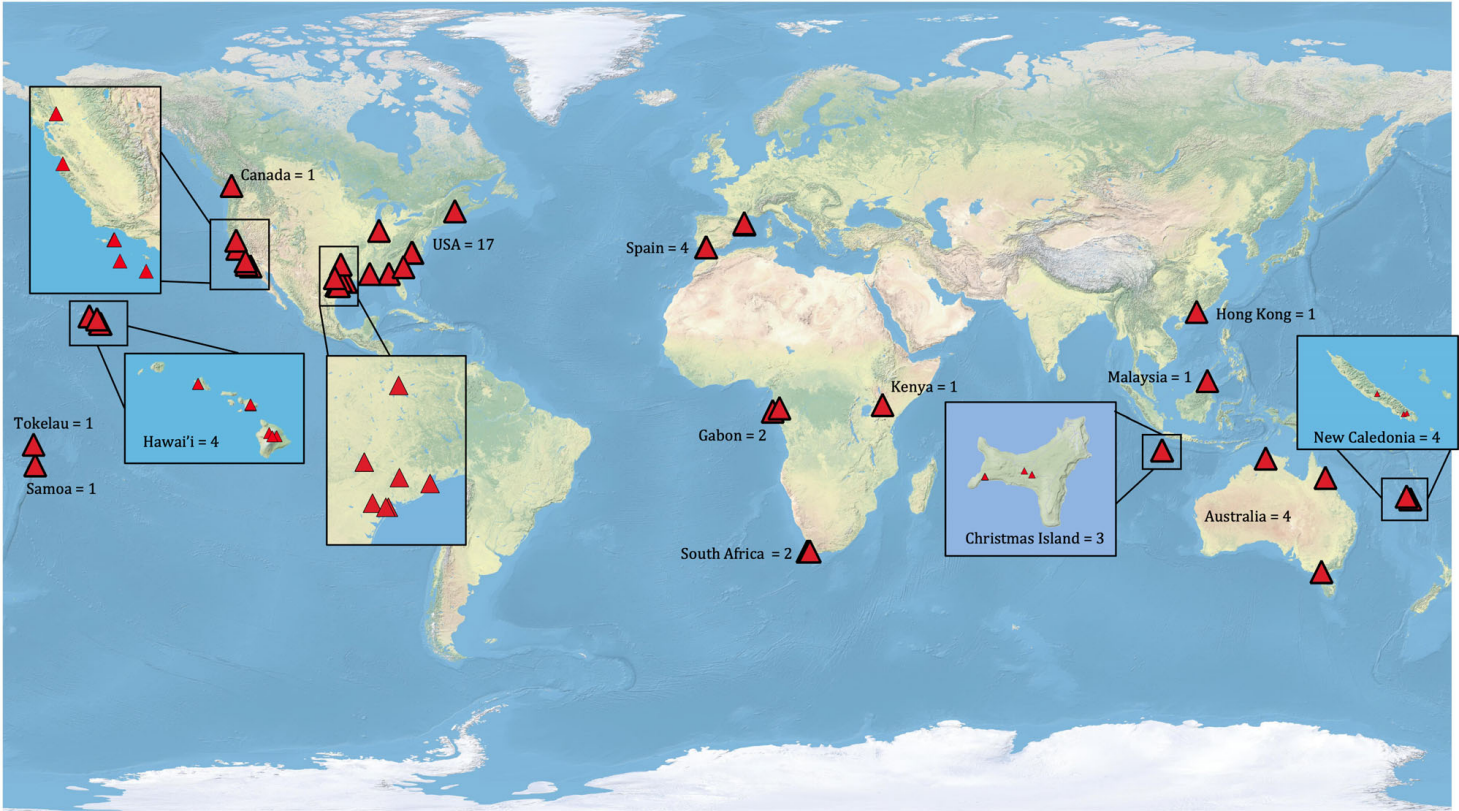
Locations of studies included in meta‐analysis. Red triangles indicate individual study sites; numbers indicate the number of articles included from each country from which data were extracted. Insets show areas where study sites are at high density. There are more study sites than articles because articles typically used several study sites. Points may completely overlap if study sites are near one another.

In total, we extracted data from 211 studies published in 46 peer‐reviewed journal articles (Figure 1), of which 53 and 156 were richness and abundance responses, respectively. We separated local taxon responses by order or class for invertebrates (e.g. Coleoptera, Chilopoda, and Araneae) and class for vertebrates (e.g. Reptilia and Amphibia). We did this because studies typically report local responses using these taxonomic groups. The exception to this is for native ants, which we separated from other Hymenoptera in all analyses because they are likely to present unique responses. If order‐ or class‐level changes to invertebrates are not reported, these are simply reported as ‘invertebrates’. In addition to invasive ant species and local taxon responses in the paired sites, we extracted the following data for each study: coordinates of study sites, country, whether the site is an island, habitat type, duration of study, sampling method, use of formicides (and active ingredient if so) and number of samples per site. All codes for these variables and the data extraction spreadsheet used to aid the data extraction process can be found in the Appendix. We also provide the full meta‐analysis protocol designed to ensure robust and repeatable results (Supplementary File). Data in tables or text were directly extracted and used. When data were expressed only graphically, we used WebPlotDigitizer to extract data values. This program can be found and downloaded here: https://automeris.io/WebPlotDigitizer/.

To ensure robust and valid data extraction, a second meta‐analyst undertook full‐text article screening and data extraction on a random subset of 28 of the 120 articles that passed the article screening stage. The second meta‐analyst was not able to screen the full selection of articles because of time constraints. This was done ‘blind’, that is, with no knowledge of the first meta‐analyst's decision to include a study or extracted data values. Article choice and data extracted were not significantly different between the two meta‐analysts (Appendix S2 and Table S2), and raw extracted data were on average 87.5% similar (range = 72–100%) for richness data and 83.5% (range = 62.1–96.1%) for abundance data. This ratified our robust data extraction protocol. Data extracted by the first analyst were therefore used in all meta‐analyses after these checks.

### Meta‐analysis

We measured the magnitude of local responses to invasion by nine non‐native ant species (*Anoplolepis gracilipes*, *Brachyponera chinensis, Linepithema humile, Myrmica rubra, Pheidole megacephala, Solenopsis invicta, Solenopsis papuana, Tapinoma sessile* and *Wasmannia auropunctata*), covering three classes of terrestrial vertebrate and a wide array of invertebrate taxa on five continents and in a range of habitats, including tropical, temperate and boreal forests and grasslands (Table S3). We did this using a standardised mean difference (SMD) approach and random‐effects models (Koricheva et al., 2013) using R package ‘metafor’ (Viechtbauer, 2010) in R version 4.2.0 (R Core Team, 2021). We chose Hedges' *g* as our effect size measurement because it is not affected by unequal sampling variances in the paired groups and includes a correction factor for small sample size (Koricheva et al., 2013). To do this, we extracted the mean species richness or abundance value in paired uninvaded and invaded sites in each study and the associated standard deviation (SD) for each mean. All studies that reported means reported either standard deviation or standard error (SE). If SE was reported rather than SD, we calculated SD using Equation (1):

(1) $$\mathrm{SD}=\mathrm{SE}\sqrt{n}$$

The Hedges' *g* value of a study was calculated using Equations (2) and (3):

(2) $$g=\frac{{\bar{x}}_{1}-{\bar{x}}_{2}}{\sqrt{\frac{\left(n_{1}-1\right)s_{1}^{2}+\left(n_{2}-1\right)s_{2}^{2}}{n_{1}+n_{2}-2}}}J,$$

where

(3) $$J=1-\frac{3}{4\left(n_{1}+n_{2}-2\right)-1}$$

is a correction for small sample size and subscripts _1_ and _2_ denote the uninvaded and invaded groups, respectively; thus, *x¯*_1_ and *x¯*_2_ are the mean local species response values across uninvaded and invaded sites, *n*_1_ and *n*_2_ denote sample size of uninvaded and invaded sites, and *s*_1_ and *s*_2_ are the standard deviations of uninvaded and invaded groups.

To compute the overall percentage change in local species richness and abundance between uninvaded and invaded sites, we first calculated the response ratio *R* and its natural logarithm using Equation (4) (Koricheva et al., 2013)

(4) $$\ln R=\ln\left(\frac{{\bar{x}}_{1}}{{\bar{x}}_{2}}\right)$$

and then the overall percentage change in abundance and richness between uninvaded and invaded sites using Equation (5)

(5) $$\left(e^{R^{+}}-1\right)\times 100$$

where *R* ^+^ is the mean response ratio (*R*) weighted by the inverse of the variance of included studies.

We separated abundance and richness responses into two analyses and used separate linear random‐effects models to determine the effect of invasive ants on local animal communities. The abundance model was based on 156 studies from 40 articles, whilst the richness model used 53 studies from 26 articles. Each ‘study’ accounted for a separate local taxon responding to non‐native ants in each article, or a conglomerate of taxa, such as ‘invertebrates’. All models weighted each study by the inverse of its variance as well as between‐study variance. Both models revealed highly heterogenous responses by local taxa (abundance: Cochran's *Q*_*M*_(df = 155) = 331.12, *p* = <0.0001, *I* ^2^ = 48.08%, *H* ^2^ = 1.93, 𝜏^2^ = 0.39; richness: Cochran's *Q*_*M*_(df = 55) = 317.38, *p* = <0.0001, *I* ^2^ = 87.88%, *H* ^2^ = 8.25, 𝜏^2^ = 2.67). We accounted for potential non‐independence of studies from each article by including article identity as a moderator variable, that is, a variable that may account for variability in the effect sizes between studies. We did this in a mixed‐effects model (random effects within subgroups, fixed effects between subgroups) (Koricheva et al., 2013) and found that article identity best explained the variance in effect sizes for both abundance and richness models (abundance: *Q*_*M*_(df = 39) = 98.56, *p* = <0.0001, *R* ^2^ = 46.3%; richness: *Q*_*M*_(df = 25) = 55.6, *p* = 0.0004, *R* ^2^ = 41.46%). We tested additional moderator variables using mixed‐effects linear models to determine whether different variables, such as invasive ant species identity, location and habitat, accounted for variability in local responses to ant invasion (Tables 1 and S4).

**TABLE 1:** Moderator analyses were conducted by running separate univariate meta‐analysis mixed‐effects models (‘Model’) to estimate whether a given variable (‘Moderator’) explained a significant or large proportion of the variation in local responses to ant invasion (‘*R* ^2^’).

Model	Moderator variable	*Q*_*M*_(df)	*p*	Sig.	*R* ^2^
Abundance by native taxon	Native taxon	65.68 (29)	0.0001	*	28.84%
Abundance by native taxon	Invasive ant species	9.26 (8)	0.32		0.00%
Abundance by native taxon	Habitat	8.3 (9)	0.5		0.00%
Abundance by native taxon	Article	98.56 (39)	0.0001	*	46.3%
Abundance by native taxon	Use of formicides	3.96 (4)	0.41		0.00%
Abundance by native taxon	Island or continental	0.16 (1)	0.69		0.00%
Richness by native taxon	Native taxon	6.3 (9)	0.70		0.00%
Richness by native taxon	Invasive ant species	23.84 (7)	0.0012	*	25.5%
Richness by native taxon	Habitat	11.36 (7)	0.12		0.00%
Richness by native taxon	Article	55.6 (25)	0.0004	*	41.46%
Richness by native taxon	Use of formicides	4.1 (3)	0.25		0.00%
Richness by native taxon	Island or continental	0.74 (1)	0.38		0.91%
Abundance by article	Invasive ant species	12.39 (8)	0.13		18.29%
Abundance by article	Habitat	8.81 (8)	0.36		0.00%
Abundance by article	Island or continental	0.22 (1)	0.63		0.00%
Richness by article	Invasive ant species	11.38 (7)	0.12		5.68%
Richness by article	Habitat	9.8 (7)	0.2		0.00%
Richness by article	Island or continental	0.014 (1)	0.71		0.00%

*Note*: Table S4 includes results for additional moderator variables that were not of direct ecological interest.

Article identity moderated the effect size more than all other tested variables, and we attributed this to the fact that each article examined a unique ecological community of interacting and dependent species. We attempted to account for this by running two additional mixed‐effects models to measure article‐level abundance and richness responses. We did this by combining raw response results for each article (the mean of individual taxon responses). On average, article‐level abundance and richness responses combined 3.88 (SD ± 4.77) and 2 (SD ± 1.92) taxon‐specific results, respectively. Both article‐level models were highly heterogenous (abundance: Cochran's *Q*_*M*_(df = 39) = 150.93, *p* = <0.0001, *I* ^2^ = 72.35%, *H* ^2^ = 3.62, 𝜏^2^ = 0.94; richness: Cochran's *Q*_*M*_(df = 25) = 166.62, *p* = <0.0001, *I* ^2^ = 86.62%, *H* ^2^ = 7.47, 𝜏^2^ = 2.2).

We verified the robustness of our meta‐analysis using the checklist of Koricheva et al. (2013), Koricheva and Gurevitch (2014). Though this checklist was designed for meta‐analyses in plant ecology, it is applicable here. All quality criteria were fulfilled (Table S5). For all four models, we conducted sensitivity analyses to explore whether the results were sensitive to publication bias, single studies skewing results or a small sample size. We first tested for whether articles were more likely to be published if they reported certain results, for example, whether negative results were less likely to be published. First, we created funnel plots to visualise the distribution of effect sizes and whether publication bias might be skewing this distribution (Figures S2–S5) using the ‘funnel’ function in metafor. We then statistically tested for publication bias with the trim‐and‐fill method using the ‘trimfill’ function and adjusted the overall effect size accordingly if publication bias was revealed. This is a nonparametric (rank‐based) method to determine to what extent extreme results might be missed because of publication bias. None of the models showed evidence of publication bias from these analyses. We also ran leave‐one‐out analyses, where each study is sequentially omitted from the results to explore to what degree the overall results depend on each individual study. No outlier studies were found. Finally, we calculated Rosenthal's Fail‐safe *N* for all models. Rosenthal's Fail‐safe *N* denotes the number of studies/articles with an effect size of zero that would need to be added to the analysis to overturn the results into non‐significance (*p* ≥ 0.05). Abundance by taxon *N* = 3888 (24.92 times the original sample size), richness by taxon *N* = 4164 (74.35 times the original sample size), abundance by article *N* = 839 (20.98 times the original sample size), richness by article *N* = 973 (37.42 times the original sample size). All functions were from the ‘metafor’ package (Viechtbauer, 2010) in R version 4.2.0 (R Core Team, 2021).

## RESULTS

**FIGURE 2:**
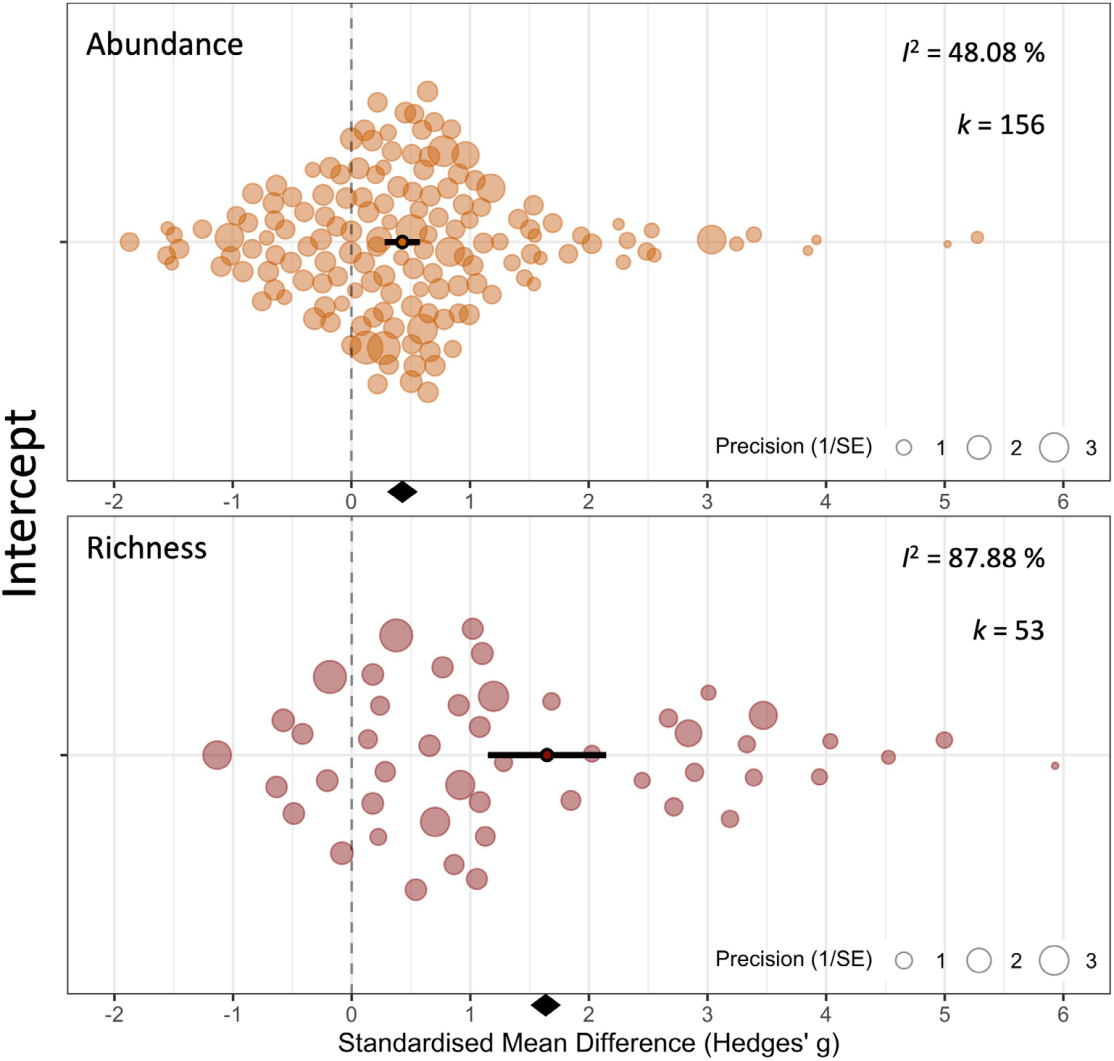
Local responses to invasive ants by taxon. The upper plot shows abundance responses, and the lower plot shows richness responses. Solid dots with black bars represent the overall standardised mean difference (Hedges' *g*) and 95% confidence intervals, respectively. Translucent circles represent individual taxon responses extracted from each article. The size of each circle is proportional to its relative weighting in the overall model and the inverse of its variance. A positive effect size means that invasive ants are reducing local diversity. The diamond at the bottom of each plot shows the overall effect size. The *k* value denotes the number of data points (‘studies’) in the model, whilst *I* ^2^ denotes the level of heterogeneity between effect sizes in the model. The position on the *y*‐axis (‘intercept’) ensures that points are visible and do not overlap.

**FIGURE 3:**
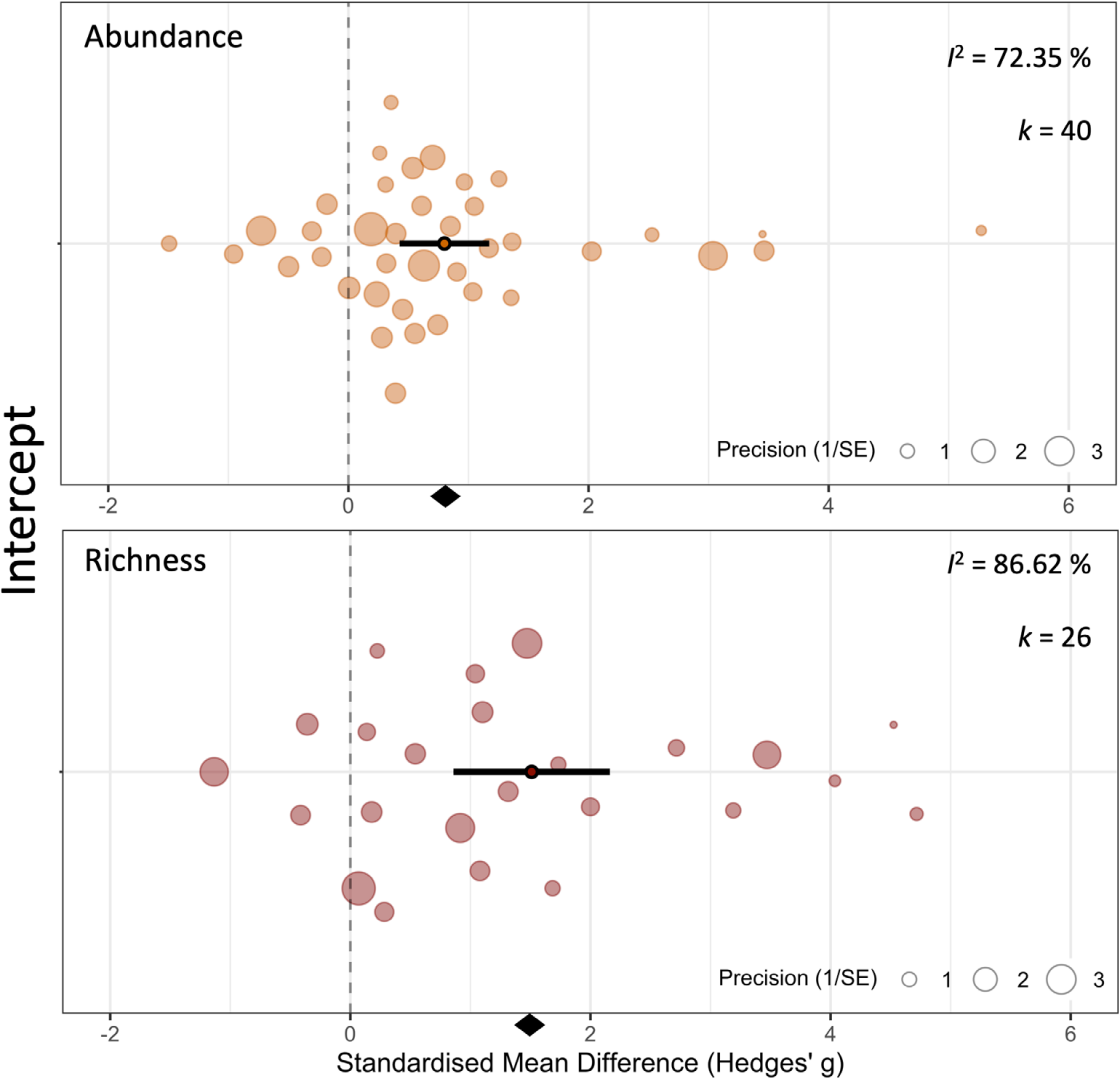
Local responses to non‐native ants by article. The upper plot shows abundance responses, and the lower plot shows richness responses. Solid dots with black bars represent the overall standardised mean difference (Hedges' *g*) and 95% confidence intervals, respectively. Translucent circles represent local community responses to invasive ants by combining taxon‐specific responses within each article. The size of each circle is proportional to its relative weighting in the overall model and the inverse of its variance. A positive effect size means that invasive ants are reducing community diversity. The diamond at the bottom of each plot indicates the overall effect size. The *k* value denotes the number of data points (‘studies’) in the model, whilst *I* ^2^ denotes the level of heterogeneity between effect sizes in the model. The position on the *y*‐axis (‘intercept’) ensures that points are visible and do not overlap.

Invasion by non‐native ants was associated with large significant decreases to local species abundance and richness across all four analyses. For analyses where articles were split into separate studies for each responding local taxon (Figure 2), both local species abundance and richness were significantly lower in areas invaded by ants (abundance response by local taxon: mean Hedges' *g* [± 95% CI] = 0.42 [0.28–0.57], *p* = <0.0001, 42.79% reduction; richness response by local taxon: mean Hedges' *g* [± 95% CI] = 1.64 [1.14–2.14], *p* = <0.0001, 53.56% reduction). Because article identity explained the greatest amount of heterogeneity in both abundance and richness models, we ran two additional meta‐analyses looking at the combined responses reported in each article (Figure 3) to account for the potential non‐independence of taxon responses in each article. These showed a similar trend, but with community abundance responses stronger and community richness approximately equal (abundance by article: mean Hedges' *g* [± 95% CI] = 0.8 [0.43–1.17], *p* = <0.0001, 52.67% reduction; richness by article: mean Hedges' *g* [± 95% CI] = 1.51 [0.86–2.16], *p* = <0.0001, 53.47% reduction). A positive effect size denotes that local taxa are lower in abundance or richness in areas invaded by ants. Cohen (1988) suggests Hedges' *g* can be interpreted as follows: 0.2 = a small effect that cannot be discerned by the naked eye; 0.5 = a medium effect; 0.8 = a large effect immediately noticeable. This rule of thumb is designed for meta‐analyses in the social sciences and therefore may be less relevant to ecological meta‐analyses.

**FIGURE 4:**
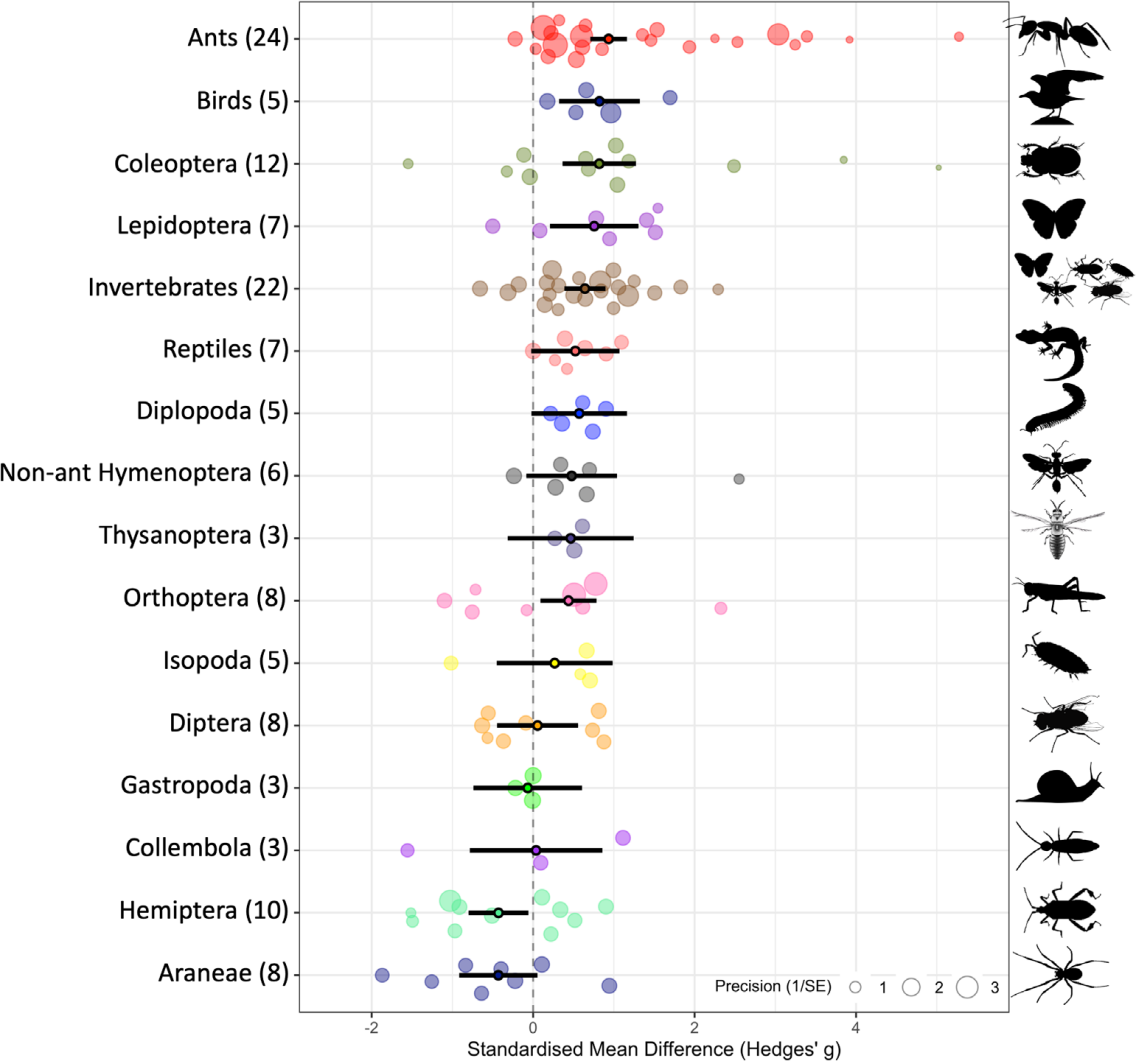
Local abundance responses to invasive ants separated by taxon. The number of studies *k* for each taxon is in parentheses by taxon labels. Solid dots with black bars represent the overall standardised mean difference (Hedges' *g*) and 95% confidence intervals, respectively. Translucent circles represent individual taxon responses. The size of each circle is proportional to its relative weighting in the overall model and the inverse of its variance. A positive effect size means that invasive ants are reducing taxon abundance. Taxa with fewer than three studies were omitted from the plot to aid visualisation. The position on the *y*‐axis (‘intercept’) ensures that points are visible and do not overlap and is also determined by the taxonomic grouping variable.

**FIGURE 5:**
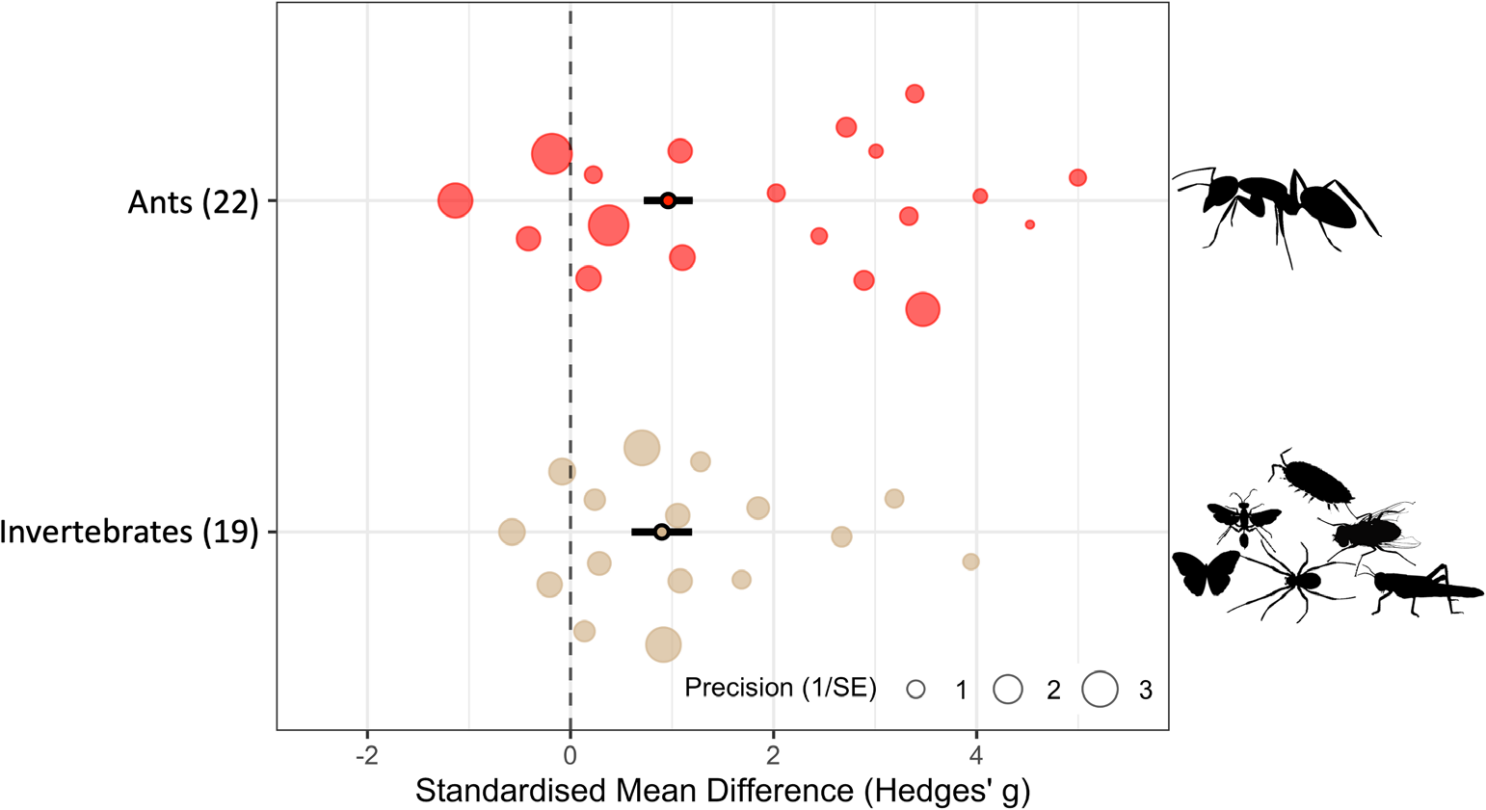
Local richness responses of ants and invertebrate communities to non‐native ants. The number of studies for each taxon is in parentheses by taxon labels. Solid dots with black bars represent the overall standardised mean difference (Hedges' *g*) and 95% confidence intervals, respectively. Translucent circles represent individual taxon responses. The size of each circle is proportional to its relative weighting in the overall model and the inverse of its variance. A positive effect size means that invasive ants are reducing taxon richness. The position on the *y*‐axis (‘intercept’) ensures that points are visible and do not overlap and is also determined by the taxonomic grouping variable. Several studies with very high effect sizes (>5) and taxa with fewer than five studies were omitted from the plot to aid visualisation of the remaining data.

Native ants showed some of the strongest negative responses of all local taxa to invasive ants (Figures 4 and 5). Where species level response data were combined by authors (termed ‘invertebrates’ in our analyses), we similarly saw strong abundance and richness responses.

## DISCUSSION

Our results show that non‐native ants severely reduce animal community diversity in relatively undisturbed natural systems across continents and habitat types. The results of this study therefore corroborate long‐held assumptions that non‐native ants may be significantly contributing to reductions in animal biodiversity globally. Moreover, our meta‐analytical design restricted studies to relatively intact areas free from other stressors, identifying non‐native ants as drivers of biodiversity change in study sites rather than passengers of other anthropogenic impacts (Stuble et al., 2013). Our results broadly conform to a previous meta‐analysis by Cameron et al. (2016), investigating the impacts of non‐native terrestrial invertebrates more generally, of which non‐native ants were a large proportion, but we found stronger impacts on community abundance (29% vs. 52.67% reduction) and richness (33% vs. 53.47% reduction). We were unfortunately unable to extract the effect of invasive ants solely from that of other non‐native taxa in their study, and thus cannot make any strong inferences about the discrepancy in the strength of the results. The key difference between the present study and that of Cameron et al.'s is that our meta‐analysis used studies conducted in intact natural areas. Of the 46 articles we selected, only 15 were shared with Cameron et al.'s study, presumably because: (1) our search was conducted 8 years later, resulting in more studies being available, (2) we disqualified many of the studies included in the Cameron et al. meta‐analysis due to our focus on undisturbed habitats and (3) our search may have more comprehensively identified studies that conformed to our specific inclusion criteria, which was focussed on capturing all studies relating to non‐native ant species rather than non‐native terrestrial invertebrates more generally.

The severity of a given response to non‐native ants appears to be primarily determined at the community‐level. Given that almost all non‐native ants are highly abundant generalist species (Holway et al., 2002; Tillberg et al., 2007), they are capable of directly and indirectly influencing a very large proportion of animal species in areas they colonise (Hölldobler & Wilson, 1990). These indirect effects may somewhat confound taxon‐specific results. For example, our analyses show that native ant diversity decreases dramatically in areas colonised by non‐native ants, but even in this group, however, some native ants appear to benefit from non‐native ants, as shown by the small number of studies showing positive responses. One possible explanation for this is that non‐native ants indirectly benefit some native species by removing their predators or competitors, for instance, and similar results may be true of other taxa. Indirect effects may have multiple levels, cascading through an ecological community in unpredictable ways and partly confounding taxon‐specific responses. Moreover, it may be the unique community composition that can determine whether the fundamental function of an ecosystem alters after non‐native ant invasion, ultimately leading to ‘invasional meltdown’ (Handler et al., 2007; O'Dowd et al., 2003; O'Loughlin & Green, 2015; Rowles & O'Dowd, 2009; Stuble et al., 2013). Such case studies unanimously detail or suggest very large direct and indirect impacts by non‐native ants.

The mechanistic underpinnings as to why native diversity falls substantially once non‐native ants have invaded are likely to be multifaceted, incorporating direct, indirect, lethal and sub‐lethal interactions. Both predatory and competitive processes appear to govern the responses of native ants to non‐native ant invasion, for example (Holway, 1999; Holway & Case, 2001; Human & Gordon, 1996; Rowles & O'Dowd, 2007; Zee & Holway, 2006). Invasive ants can ‘break’ the discovery‐dominance trade‐off thought to structure many native ant assemblages (Arnan et al., 2018; Bertelsmeier et al., 2015; though see Parr & Gibb, 2012), ultimately allowing them to dominate food resources to such an extent that native ants are unable to coexist. Invasive ants are typically also hyper‐abundant because of their ability to control resources and their release from natural enemies and strong competitive forces (Porter et al., 1997). Most research identifying the mechanisms behind diversity declines relates to native ants responding to invasive ants. Our results suggest that entire communities of disparate animal taxa respond negatively to non‐native ants, and the mechanistic cause of these declines may vary between communities and taxa. Generating accurate species‐level interaction data of invasive ants using high‐throughput DNA‐based methods (e.g. dietary metabarcoding), for example, could help pinpoint the mechanisms behind certain taxon or community responses. These methods could reveal competition for food resources between invasive ants and native species or if predation of particular groups during the initial stages of invasion might be the cause of diversity declines. Research generating species‐level interaction data of invasive ants paired with surveys of native diversity at different stages of invasion therefore merits further exploration.

Our results also show some trends in taxon‐specific responses to non‐native ants. Hemiptera was the only group to show significant increases in abundance in invaded zones. Ants defend exudate‐producing insects (e.g. aphids, scale insects) from natural enemies in return for honeydew, a carbohydrate‐rich excretion (Stadler & Dixon, 2005). High densities of non‐native ants in invaded zones may be more effective at defending these hemipterans from predators and parasitoids (Holway et al., 2002; Kaplan & Eubanks, 2005; Styrsky & Eubanks, 2007). In contrast, native ants, birds, reptiles, beetles and Lepidoptera all show very strong negative abundance responses to non‐native ants overall. These results might be useful for conservation managers aiming to protect certain threatened species or communities. Furthermore, the conglomerate group ‘invertebrates’ responded strongly to non‐native ants, providing clear evidence that non‐native ants can deconstruct and diminish invertebrate communities in relatively natural systems (Berman et al., 2013; Rowles & O'Dowd, 2009). Invertebrates undertake and contribute to a large proportion of ecosystem processes (Prather et al., 2013); significant declines in invertebrate diversity could therefore substantially affect the wider functioning of the ecosystem.

Though we tested for several sources of bias, which were not found, some limitations may remain. For example, it was difficult to measure sampling effort between studies because collection methods varied considerably between articles within a sampling type (e.g. pitfall traps used in one study may have had a larger aperture, greater volume or a different design than those used in another study). This lessened our ability to compare study reliability and scale at the level of individual traps. Moreover, some of the studies we included in our analyses investigating local invertebrate responses to invasive ants may not have been able to accurately distinguish whether all invertebrates captured were native. Some invertebrates in these communities may have been non‐native, which could confound results. Our conclusion that we are observing native communities responding to invasive ants is greatly strengthened because of the stringent eligibility criteria we applied that disqualified studies where sites were considered ‘degraded’; 46% of the 347 articles screened at the abstract or full‐text stage were disqualified because they violated these conditions. However, even in otherwise ‘intact’ habitats, non‐native species are often present, though typically in low numbers. Ultimately, this is a variable we cannot completely control for in our meta‐analysis given that many included studies did not discuss this issue. Therefore, the invertebrate communities in our analyses should be viewed as predominantly native, potentially with low abundances of non‐native species in some sites. See Appendix S3 for a discussion of further limitations.

These trends raise serious concerns about the future and long‐term existence of endemic species in natural systems where ants are invading. Invasive species are currently the second largest threat to biodiversity after land‐use change (Clavero & Garcia‐Berthou, 2005; Luque et al., 2014; Simberloff et al., 2013), and it is therefore critical to identify the specific impacts of invasive taxa in natural areas. We observe that invasive ants are a high‐risk group, posing a serious threat to native species in relatively intact native habitats. Natural systems typically hold higher overall species richness than degraded habitats and associated native communities react more strongly and more predictably to ant invasion than non‐native species in the same system (Krushelnycky & Gillespie, 2008). This synthesis suggests there are crucial considerations for conservation policy. However, solutions to the problems posed by invasive ants are not straightforward; ill‐conceived control campaigns may do more harm than good in certain ecological contexts. Though there have been many ant eradication attempts, less than half are successful and most are extraordinarily costly to employ financially and logistically for conservation managers considering the large land areas many invasive ants have colonised. For example, eradication regimes cost on average $2885 and $822 per ha for aerial and hand toxin broadcast methods, respectively (Hoffmann et al., 2016). Properly designed and resourced measures to detect and prevent the further spread of invasive ants are, nevertheless, urgently required in addition to effective control strategies for non‐native ants that have already colonised native areas of conservation concern. For example, improving the inspection process of living plants in international shipments (McGlynn, 1999) or scaling up biomonitoring of invasive species using new technologies such as eDNA or chemical approaches (Larson et al., 2020).

This study presents clear evidence showing that non‐native ants are the drivers of strong biodiversity declines at the taxon‐ and community‐level across multiple habitats and geographical locations around the world. These impacts affect both vertebrate and invertebrate taxa. The responses are observed in relatively undisturbed environments where habitats consist entirely or almost entirely of native plant species, showing that the impact of non‐native ants is not limited to disturbed habitats. Reductions to animal community biodiversity may have severe consequences for ecosystem functioning and the long‐term future of endemic species.

## AUTHOR CONTRIBUTIONS

**Maximillian Tercel PTG:** Conceptualization; investigation; writing – original draft; methodology; validation; visualization; writing – review and editing; software; formal analysis; data curation. **Jordan Cuff P:** Investigation; writing – review and editing; visualization; methodology. **William Symondson OC:** Funding acquisition; writing – review and editing; validation; project administration; supervision. **Ian Vaughan P:** Funding acquisition; writing – review and editing; validation; methodology; investigation; formal analysis; project administration; supervision.

## CONFLICT OF INTEREST STATEMENT

The authors declare no conflicts of interest.

## Supporting information


**Appendix S1.** Supporting Information.

**Data S1.** Supporting Information.

**Data S2.** Supporting Information.

**Data S3.** Supporting Information.

**Data S4.** Supporting Information.

## Data Availability

A full list of screened articles, excluded article references with reasons for exclusion, included articles and raw extracted data are available as supplementary files. Code for statistical analysis is available as a supplementary file.
