# Supplementary material for: Non‐native ants drive dramatic declines in animal community diversity: A meta‐analysis
Source: Insect Conserv Divers. 2023 Jul 29;16(6):733–44. doi: 10.1111/icad.12672 (PMC10947240; doi:10.1111/icad.12672)
Supplement: Supplementary file 1 — Appendix S1. Supporting Information. [file ICAD-16-733-s002.docx]

Appendix S1

*S1 Article search*

Boolean search string used in [www.webofscience.com](http://www.webofscience.com) “topic search”:

*TS=(("Pheidole megacephala" OR "African big-headed ant*" OR "Anoplolepis gracilipes" OR "Yellow crazy ant*" OR "Solenopsis invicta" OR "red imported fire ant*" OR "Myrmica rubra" OR "European fire ant*" OR "Linepithema humile" OR "Argentine ant*" OR "Wasmannia auropunctata" OR "little fire ant*" OR "electric ant*" OR "Paratrechina longicornis" OR "longhorn crazy ant*" OR "black crazy ant*" OR "Technomyrmex albipes" OR "white footed ant*" OR "white-footed ant*" OR "Brachymyrmex cordemoyi" OR "Nylanderia bourbonica" OR "Trichnomyrmex destructor" OR "Solenopsis geminata" OR "tropical fire ant*" OR "Tapinoma melanocephalum" OR "ghost ant*" OR "invasive ant" OR "invasive ants" OR "exotic ant" OR "exotic ants" OR "introduced ant" OR "introduced ants" OR "alien ant" OR "alien ants" OR "invading ants" OR "pest ants" OR "tramp ants" OR "tramp ant")*

*AND*

*(abundance OR divers* OR biodiversity OR richness OR community OR communities)*

*NEAR/5*

*(impact* OR effect* OR influence OR influences OR affect* OR damag* OR consequence* OR decline* OR increas* OR decreas* OR replace* OR destruction OR introduc* OR invasi* OR respon*))*

This returned 800 articles on 30^th^ January, 2023. We chose to include only articles in English because we were unable to include collaborators sufficiently fluent in other languages. We also emailed Dr. Ben Hoffmann, an expert in invasive ant ecology, asking if they knew of any sources of grey literature or unpublished studies that might adhere to our selection criteria but they were not aware of any sources of additional literature. We paired this with extensive internet searches in non-academic search engines (e.g. Google), but revealed no additional sources of primary data.

Figure S1. PRISMA flow diagram of article screening, eligibility scrutiny, and inclusion in meta-analysis.

Records excluded
from title = 453; from abstract = 227; total excluded before searching full-text = 680)

Records screened
(n = 800)

Records after duplicates removed
(n = 800)

Studies included in quantitative synthesis (meta-analysis)
(n = 46)

Full-text articles excluded with reasons
(n = 74)

Included

Eligibility

Screening

Identification

Full-text articles assessed for eligibility
(n = 120)

Studies included in qualitative synthesis
(n = 46)

Additional records identified through other sources
(n = 0)

Records identified through database searching
(n = 800)

Table S1. Data extraction sheet used to aid data collection. Acc# denotes the accession number generated for an article. Authors, Date, Title, denote journal information; Invasive_ant denotes the focal species of introduced ant in a given study; Native_taxon denotes the native taxon responding to the introduced ant; Coordinates and Location relate to the geography of the study sites; Habitat denotes the primary habitat type of a site; Duration denotes the total sampling duration across months or years; Sampling_method denotes the type of sampling used to sample the given native taxon; Formicides denotes whether toxic formicides are used and the active ingredient if so; Page_of_acc is the page of the article data were extracted from; Parameter is either abundance or richness; Data_source is either Table(number), Figure(number), or text and denotes where values were extracted from in an article; X_uninv, SD_uninv, and N_uninv denotes the mean, standard deviation, and sample size of the native taxon parameter measurement in uninvaded sites; X_invade, SD_invade, and N_invade denotes the native taxon parameter measurement in invaded sites. Samples_per_site denotes the number of samples used per site; Comments provides the meta-analyst with additional space to make any further comments.

| 1. Acc# | 2. Authors | 3. Date | 4. Title | 5. Invasive_ant |
| --- | --- | --- | --- | --- |
|  |  |  |  |  |
| 6. Native_taxon | 7. Coordinates | 8. Location | 9. Habitat | 10. Duration |
|  |  |  |  |  |
| 11. Sampling_method | 12. Formicides | 13. Page_of_acc | 14. Parameter | 15. Data_source |
|  |  |  |  |  |
| 16. X_uninv | 17. SD_uninv | 18. N_uninv | 19. X_invade | 20. SD_invade |
|  |  |  |  |  |
| 21. N_invade | 22. Samples_per_site | 23. Comments |  |  |
|  |  |  |  |  |

*S2 Second meta-analyst*

To ensure robust and valid data extraction, a second meta-analyst undertook full-text article screening and data extraction on a random subset of one-quarter of the articles. The second meta-analyst did not screen the full selection of articles because of time-constraints. This was conducted “blind”, i.e., without knowledge of the results or articles selected by the first meta-analyst. Wilcoxon rank sum tests, non-parametric equivalents to unpaired t-tests, revealed no significant differences between calculated effect sizes or extracted data from the articles between the two meta-analysts (Table S2). Subsequent to these tests, any disagreements in article inclusion or notable differences in extracted data values were discussed between the analysts. These discussions then informed which studies in the subset would be included or disqualified. Two additional articles were included, whilst three were excluded during this step and both analysts agreed on the final list of included articles. Given that these tests ratify our robust and valid data extraction process, data extracted by the first analyst were used in all meta-analyses after these checks.

Table S2. Results of Wilcoxon rank sum tests to determine whether data or effect sizes were significantly different between the two meta-analysts.

| Variable | Invasion status | Data type | *Z* | *p* |
| --- | --- | --- | --- | --- |
| Abundance | Uninvaded | Mean | 540.5 | 0.52 |
| Abundance | Uninvaded | Standard deviation | 418.5 | 0.31 |
| Abundance | Uninvaded | Sample size | 379.5 | 0.1 |
| Abundance | Invaded | Mean | 530.5 | 0.61 |
| Abundance | Invaded | Standard deviation | 466.5 | 0.72 |
| Abundance | Invaded | Sample size | 364 | 0.061 |
| Richness | Uninvaded | Mean | 33 | 0.96 |
| Richness | Uninvaded | Standard deviation | 28 | 0.71 |
| Richness | Uninvaded | Sample size | 29 | 0.7 |
| Richness | Invaded | Mean | 30 | 0.87 |
| Richness | Invaded | Standard deviation | 28 | 0.71 |
| Richness | Invaded | Sample size | 29 | 0.7 |
| Abundance | - | Hedges’ *g* | 489 | 0.96 |
| Richness | - | Hedges’ *g* | 40 | 0.44 |

| Possible effect moderator | Example codes |
| --- | --- |
| Invasive ant taxon | Linepithema humile, Anoplolepis gracilipes, Brachyponera chinensis, Pheidole megacephala, Tapinoma sessile, Wasmannia auropunctata  Solenopsis invicta, Solenopsis papuana, Myrmica rubra |
| Native taxon | Acari, Amphibians, Amphipoda, Ants, Arachnida, Araneae, Birds, Blattodea, Chilopoda, Coleoptera, Collembola, Decapoda, Dermaptera, Diplopoda, Diptera, Embioptera, Gastropoda, Hemiptera, Hymenoptera, Invertebrates, Isopoda, Lepidoptera, Orthoptera, Pscoptera, Pseudoscorpiones, Reptiles, Thysanoptera, Vertebrates |
| Location | Australia, USA, Spain, New Zealand, France (New Caledonia), Malaysia, USA (Santa Cruz Island), Samoa, New Zealand (Tokelau), South Africa, USA (Hawai'i), Australia (Christmas Island), Gabon, Kenya |
| Habitat type | Coniferous forest, deciduous forest, scrubland, tropical forest, grassland, shrub, coastal scrub, littoral forest, mixed forest, mixed habitats |
| Sampling method | Litter extraction, pitfalls, foliage beats, visual surveys, foliage removal, bait transect, hand sampling, pan traps, light trap, cover board, drift fence |
| Use of formicides | No, yes (“active ingredient”) |

Table S3. Examples of possible effect size moderators coded for during data extraction and example codes for each.

Table S4. Each ‘moderator’ is a variable that could potentially influence effect sizes. We ran univariate moderator analyses in our mixed-effects meta-analysis to determine to what extent each variable affected local richness and abundance responses to invasive ants. This table includes the results of all moderator variables tested, whilst Table 1 in the main text includes only the moderator variables of direct ecological interest.

| Model | Moderator variable | *Q_M_*(df) | *p* | Sig. | *R*^2^ |
| --- | --- | --- | --- | --- | --- |
| Abundance by native taxon | Native taxon | 65.68 (29) | 0.0001 | * | 28.84% |
| Abundance by native taxon | Invasive ant species | 9.26 (8) | 0.32 |  | 0.00% |
| Abundance by native taxon | Habitat | 8.3 (9) | 0.5 |  | 0.00% |
| Abundance by native taxon | Article | 98.56 (39) | 0.0001 | * | 46.3% |
| Abundance by native taxon | Use of formicides | 3.96 (4) | 0.41 |  | 0.00% |
| Abundance by native taxon | Sampling method | 30.19 (22) | 0.11 |  | 0.00% |
| Abundance by native taxon | Latitude | 0.28 (1) | 0.59 |  | 0.00% |
| Abundance by native taxon | Longitude | 0.0022 (1) | 0.96 |  | 0.00% |
| Abundance by native taxon | Location | 30.94 (12) | 0.002 | * | 6.19% |
| Abundance by native taxon | Island or continental | 0.16 (1) | 0.69 |  | 0.00% |
| Richness by native taxon | Native taxon | 6.3 (9) | 0.70 |  | 0.00% |
| Richness by native taxon | Invasive ant species | 23.84 (7) | 0.0012 | * | 25.5% |
| Richness by native taxon | Habitat | 11.36 (7) | 0.12 |  | 0.00% |
| Richness by native taxon | Article | 55.6 (25) | 0.0004 | * | 41.46% |
| Richness by native taxon | Use of formicides | 4.1 (3) | 0.25 |  | 0.00% |
| Richness by native taxon | Sampling method* | 30.52 (19) | 0.045 | * | 24.54% |
| Richness by native taxon | Latitude | 0.53 (1) | 0.47 |  | 0.00% |
| Richness by native taxon | Longitude | 0.01 (1) | 0.75 |  | 0.00% |
| Richness by native taxon | Location**^†^** | 23.41 (12) | 0.024 | * | 11.85% |
| Richness by native taxon | Island or continental | 0.74 (1) | 0.38 |  | 0.91% |
| Abundance by article | Invasive ant species | 12.39 (8) | 0.13 |  | 18.29% |
| Abundance by article | Habitat | 8.81 (8) | 0.36 |  | 0.00% |
| Abundance by article | Latitude | 0.15 (1) | 0.7 |  | 0.00% |
| Abundance by article | Longitude | 0.28 (1) | 0.59 |  | 0.00% |
| Abundance by article | Location | 16.01 | 0.19 |  | 3.43% |
| Abundance by article | Island or continental | 0.22 (1) | 0.63 |  | 0.00% |
| Richness by article | Invasive ant species | 11.38 (7) | 0.12 |  | 5.68% |
| Richness by article | Habitat | 9.8 (7) | 0.2 |  | 0.00% |
| Richness by article | Latitude | 0.11 (1) | 0.73 |  | 0.00% |
| Richness by article | Longitude | 0.13 (1) | 0.71 |  | 0.00% |
| Richness by article | Location | 11.72(10) | 0.3 |  | 0.00% |
| Richness by article | Island or continental | 0.014 (1) | 0.71 |  | 0.00% |

*The results once a single outlier study from Porter and Savignano (1990) with a large effect size is excluded: Q_M_(df = 18) = 19.8164, p = 0.3433, R^2^ = 3.11 %.

^†^The results once a single outlier study from Naumann and Higgins (2015) with a large effect size is excluded: Q_M_(df = 11) = 11.3655, p = 0.4132, R^2^ = 0.00 %.

Table S5. Quality criteria checklist derived from Koricheva and Gurevitch, 2014

| Quality criteria | Current study |
| --- | --- |
| 1. Has formal meta-analysis been conducted (i.e. combination of effect sizes using standard meta-analytical methodology) or is it simply a vote count? | Yes, formal meta-analysis is conducted here, using a standardised mean difference approach to measure effect size. Vote counting was not used. |
| 2. Are details of bibliographic search (electronic data bases used, keyword combinations, years) reported in sufficient detail to allow replication? | Yes, all extracted data and the reference list are provided, including a full list of screened articles. A PRISMA flow diagram is also provided (Figure S2.1). |
| 3. Are criteria for study inclusion/exclusion explicitly listed? | Yes, all criteria are listed in the Methods section. Also, a full protocol for this meta-analysis is given. |
| 4. Have standard metrics of effect size been used or, if nonstandard metrics have been employed, is the distribution of these parameters known and have the authors explained how they calculated variances for such metrics? | Yes, a standard metric of effect size is employed here – standardised mean difference (Hedges’ *g* in this case). |
| 5. If more than one estimate of effect size per study was included in the analysis, has potential non-independence of these estimates been taken into account? | We tested for non-independence of effect sizes and found that there was a strong significant non-independence between effect sizes estimated from the same articles. We attributed these differences to the highly variable and unique ecological communities examined in each article that responded to introduced ants; i.e. ecological communities are themselves interdependent and this was evidenced in our additional analyses of article-level effect sizes. |
| 6. Have effect sizes been weighted by study precision or has the rational for using unweighted approach been provided? | Yes, effect sizes were weighted by variance. |
| 7. Have statistical model for meta-analysis and the software used been described? | Yes, a random-effects model (due to significant heterogeneity) were run using the “metafor” package (Viechtbauer 2010) in R version 3.6.1 (R Core Team 2014). |
| 8. Has heterogeneity of effect sizes between studies been  quantified? | Yes, heterogeneity statistics are reported (p-values, *I^2^, H^2^, tau^2^,* for each model), and confidence intervals are given for total effect size and for each study. |
| 9. Have the causes of existent heterogeneity in effect sizes been explored by meta-regression? | Yes, changes to effect size due to different variables were investigated. See Table 2.1 in main Chapter 2 text. |
| 10. If effects of multiple moderators have been tested, have  potential non-independence of and interactions between  moderators been taken into account? | Effect sizes of studies originating from the same article were combined for an overall article effect size to test for non-independence. We also tested for interactions between moderators, but none were significant. |
| 11. If meta-analysis combined studies conducted on different species, has phylogenetic relatedness of species been taken into account? | Yes, this is a secondary question this systematic review aims to answer. Relatedness of species was taken into account and analysed from the results of the meta-analysis. |
| 12. Have tests for publication bias been conducted? | Yes, funnel plots were generated and point distribution asymmetry tests (and subsequent corrections) were conducted (see Figures S2 -S5 below). |
| 13. If meta-analysis combines studies published over considerable time span, have possible temporal changes in effect size been tested? | Yes, meta-regression was used to test whether effect size of studies varied with publication date. |
| 14. Have sensitivity analysis been performed to test the  robustness of results? | Yes, a leave-one-out analysis was run to test the robustness of the overall results. |
| 15. Have full bibliographic details of primary studies included in a meta-analysis been provided? | Yes, a full list of all screened and used articles are included as a supplementary file. Excluded articles are given with a reason for exclusion. |
| 16. Has the data set used for meta-analysis, including effect sizes and variances/sample sizes from individual primary studies and moderator variables, been provided as electronic appendix? | Yes, all data have been uploaded as a supplementary file. |

Figure S2. Native species abundance response effect size and standard error per study (n = 156), used to test for publication bias. Zero additional studies are estimated to be required on the left side (SE = 7.18).


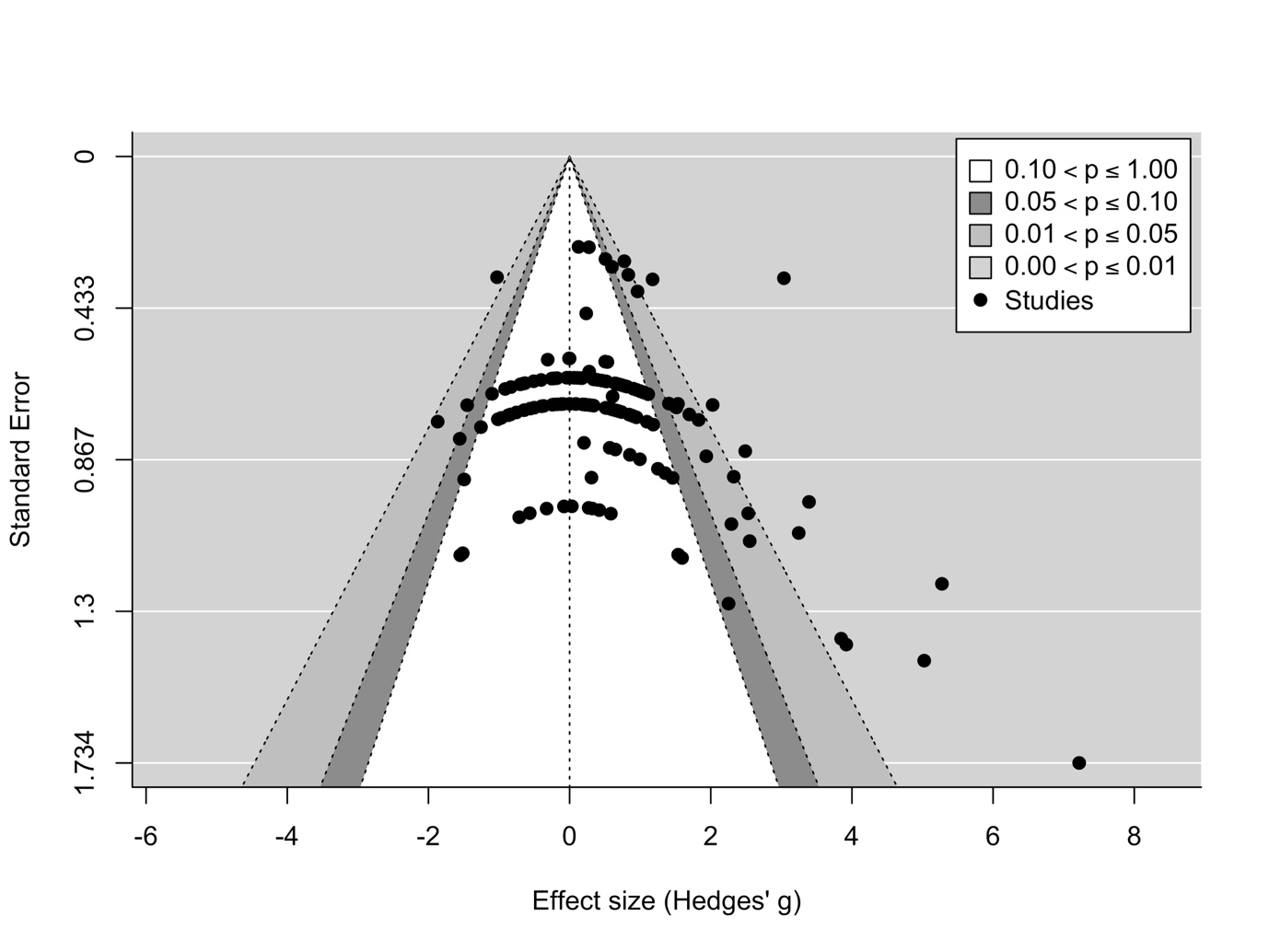


Figure S3. Native species richness response effect size and standard error per study (n = 53), used to test for publication bias. Zero additional studies are estimated to be required on the left side (SE = 4.17).


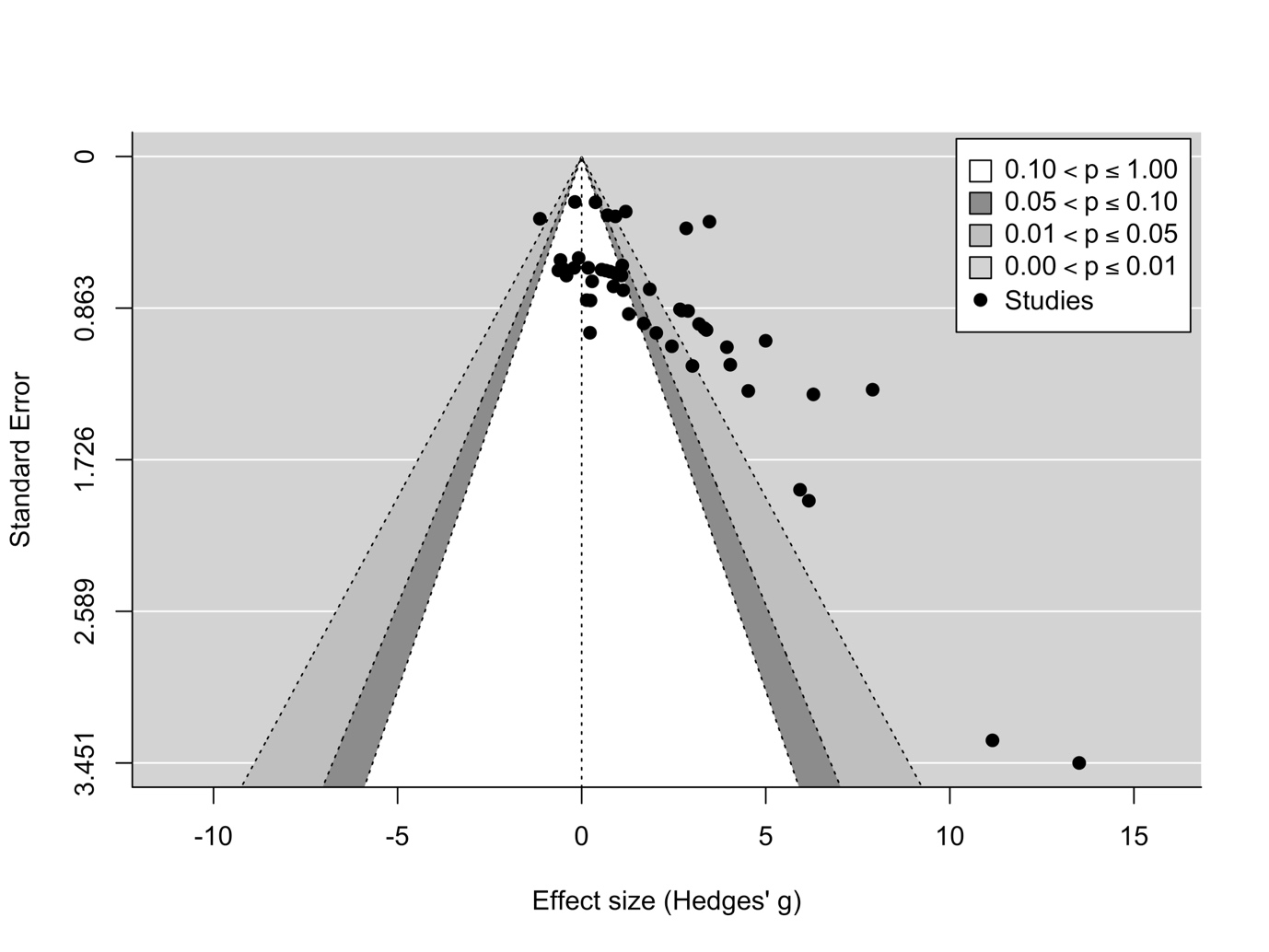


Figure S4. Community abundance response effect size and standard error per study (n = 40), used to test for publication bias. Zero additional studies are estimated to be required on the left side (SE = 3.1).


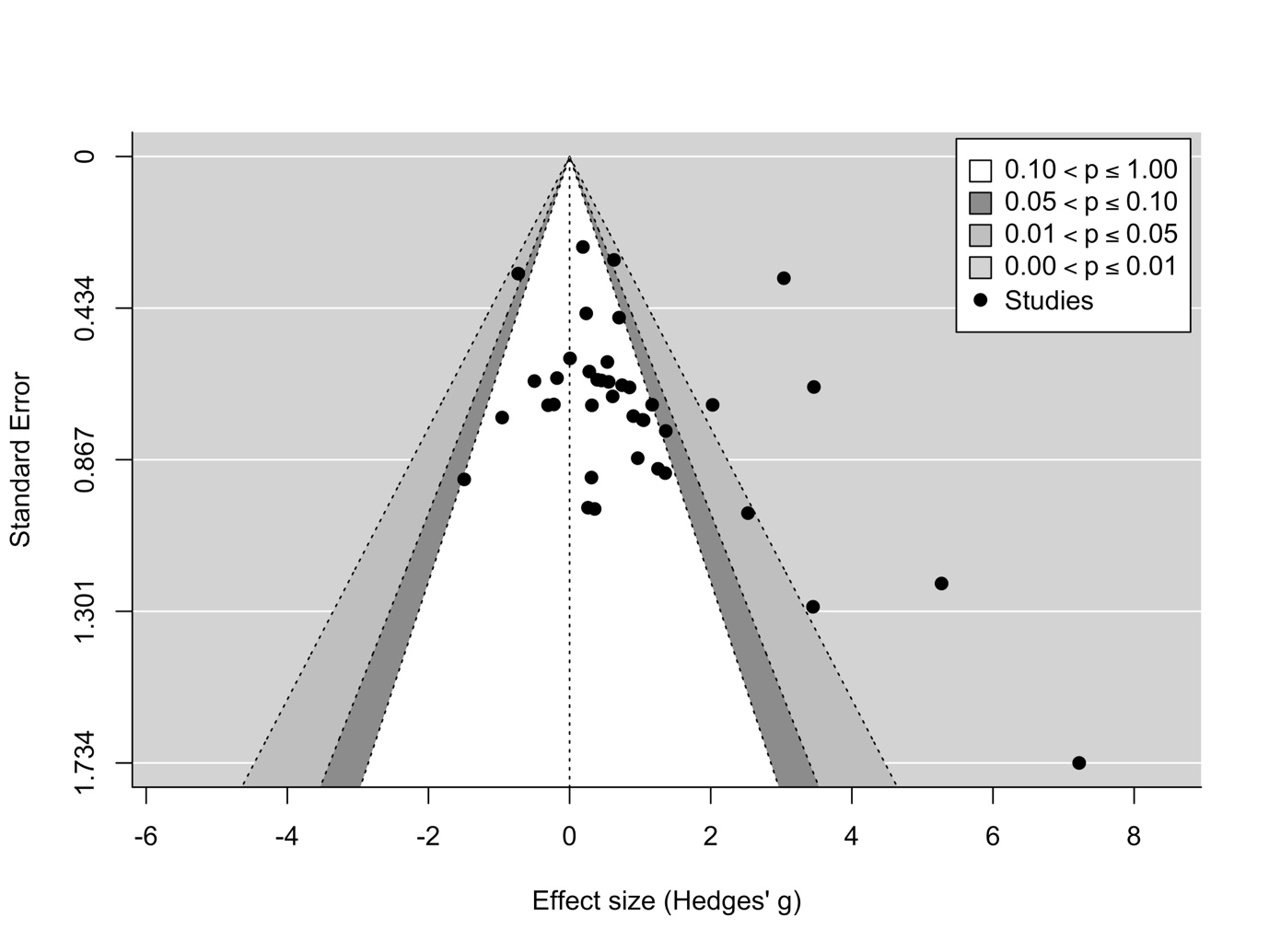


Figure S5. Community richness response effect size and standard error per study (n = 26), used to test for publication bias. Zero additional studies are estimated to be required on the left side (SE = 3.13).


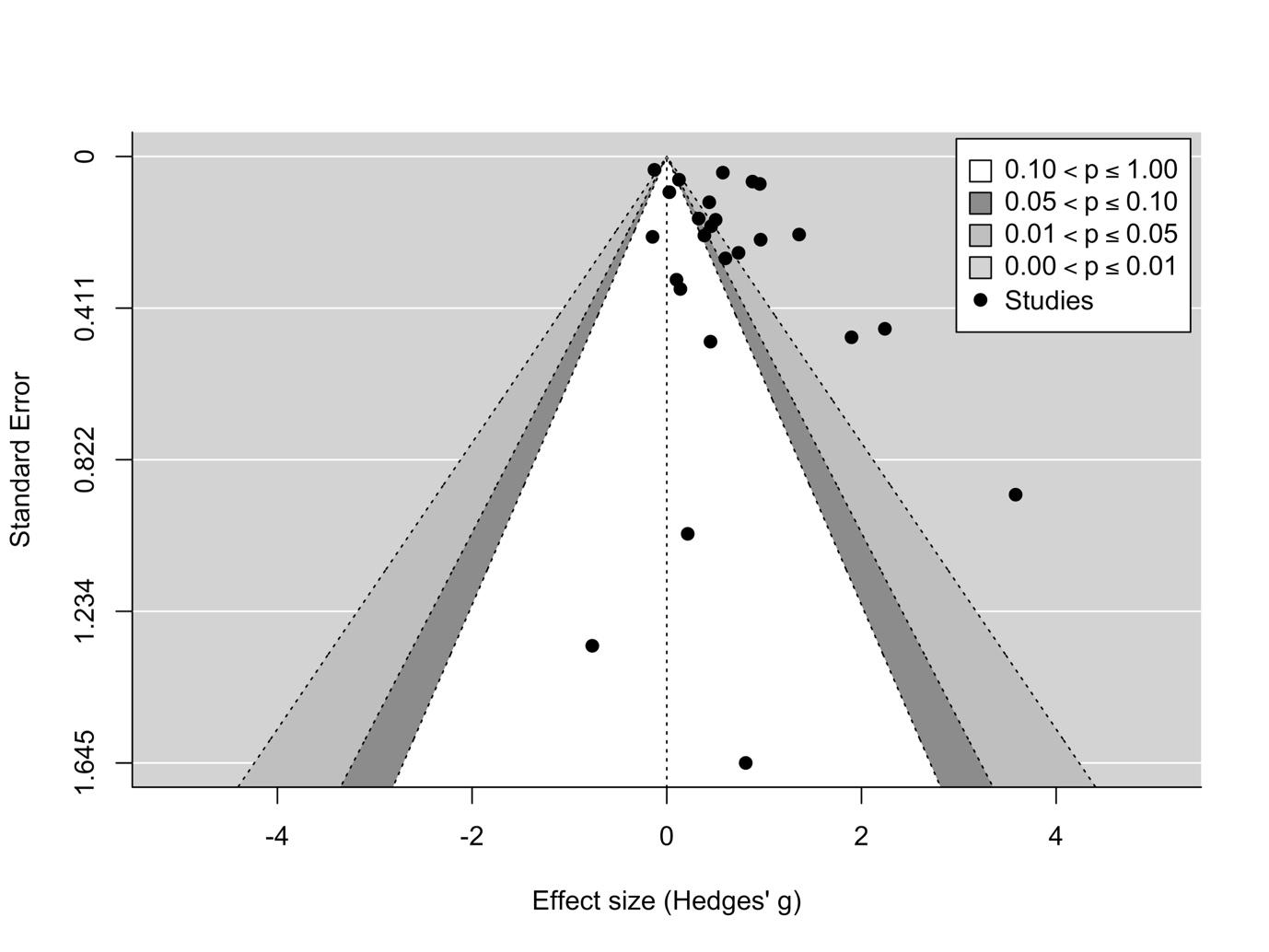


*S3 Limitations of the meta-analysis*

Though we took many steps to ensure this analysis was as robust and reliable as possible, several limitations remain (see the main text discussion for tests of bias). One key issue is that it was extremely difficult to measure the external validity of screened articles. This is primarily because sampling strategies varied so widely that sample and site numbers were not representative of a consistent “value”. For example, though one study may have used 120 total pitfall traps spread across 5 paired sites (12 traps per site), and another study may have used 150 traps across 5 paired sites (15 traps per site), the trap volume, aperture diameter, period of time left active, whether they were baited, trap spacing or layout, and the total site area may all be different. These values were not typically reported either. Nevertheless, we attempted to quantify article validity by proxy using “traps per site”. We also separated each trapping type (pitfalls, pan traps, visual surveys etc.) and analysed these separately and together. These were all non-significant effect size moderators across all analyses.

It was difficult to incorporate how different variables may interact to influence effect sizes of local response to ant invasion. Unfortunately, the subgroup sample sizes were too low for us to be able to confidently interpret the results of these models. For example, the subgroup sample size was too low to sufficiently test whether different taxa responded to invasion differently between locations or with varying latitude, which may have been a point of interest to conservationists.

Another key limitation is the lack of non-English articles, which may geographically bias results to Anglophone countries, or countries where English is typically learned/used in scientific publishing. It may be that there are biogeographical differences between regions we were unable to identify because of this patchy global coverage. Notably, several ecoregions are absent from our analysis, perhaps because of this language issue or because studies have not been undertaken in those places.

As previously acknowledged, some of the studies we included in our analysis investigating local invertebrate responses to invasive ants may not have been able to accurately distinguish whether all invertebrates captured were native. Some invertebrates in these communities may have been non-native, which could confound results. Our inference that primarily native communities are responding to invasive ants is greatly strengthened because of the stringent eligibility criteria we applied that disqualified studies where sites were considered ‘degraded’ (e.g., within or adjacent to anthropogenically modified habitats, presence of non-native plants or animals as noted by the authors of each study); 46% of the 347 articles screened at the abstract or full-text stage were disqualified because they violated these conditions explicitly or implicitly. However, even in otherwise ‘intact’ habitats, non-native species are often present, though typically in low numbers. Ultimately, this is a variable we cannot completely control for in our meta-analysis given that many included studies did not discuss this issue. Therefore, the invertebrate communities in our analysis should be viewed as predominantly native, with low abundances of non-native species.

Our sensitivity analyses did not detect any biases, but some may nevertheless remain. For example, responses to invasive ants may have been artificially inflated in studies which were purposefully carried out in areas where there was a prior knowledge of extreme declines in native species abundance or richness because of invasive ants. This is unfortunately impossible to reliably identify and could theoretically be detected from the sensitivity analyses conducted. As such, the results of some studies we used may present worst-case-scenarios. In contrast, there were several regions completely invaded by non-native ants and thus no comparisons could be made; some of these may be communities that have responded most strongly to invasion. Another potential source of bias could arise from where the local taxa studied follow the taxonomic expertise of the researchers. Response measurements may not include typically overlooked groups even if they are present and responding. This may conceal how certain components of the local communities react to ant invasion and biases observed community responses towards those of “charismatic” taxa.

For future syntheses, ideally a second (or third!) meta-analyst will conduct screening, data extraction and analyses on the full set of returned articles instead of only one-quarter. This will further improve the robustness and repeatability of results.

*S4 Bibliography of included articles* (n = 46)

Allen, C. R., Birge, H.E., Slater, J. and Wiggers, E. (2017). The invasive ant, Solenopsis invicta, reduces herpetofauna richness and abundance. Biological Invasions 19(2):713–722. doi: https://doi.org/10.1007/s10530-016-1343-7.

Allen, Craig R, Lutz, R.S. and Demarais, S. (2017). Red Imported Fire Ant Impacts on Northern Bobwhite Populations Author ( s ): Craig R . Allen , R . Scott Lutz and Stephen Demarais Published by : Wiley on behalf of the Ecological Society of America Stable URL : http://www.jstor.org/stable/1941972 JSTOR i. 5(3):632–638.

Allen, C.R., Lutz, R.S., Lockley, T., Phillips, S.A. and Demarais, S. (2001). The non-indigenous ant, Solenopsis invicta, reduces loggerhead shrike and native insect abundance. Journal of Agricultural and Urban Entomology 18(4):249–259.

Alvarez-Blanco, P., Caut, S., Cerdá, X. and Angulo, E. (2017). Native predators living in invaded areas: responses of terrestrial amphibian species to an Argentine ant invasion. Oecologia 185(1):95–106. doi: https://doi.org/10.1007/s00442-017-3929-x.

Berman, M., Andersen, A.N. and Ibanez, T. (2013). Invasive ants as back-seat drivers of native ant diversity decline in New Caledonia. Biological Invasions 15(10):2311–2331. doi: https://doi.org/10.1007/s10530-013-0455-6.

Le Breton, J., Chazeau, J. and Jourdan, H. (2003). Immediate impacts of invasion by Wasmannia auropunctata (Hymenoptera: Formicidae) on native litter ant fauna in a New Caledonian rainforest. Austral Ecology 28(2):204–209. doi: https://doi.org/10.1046/j.1442-9993.2003.01266.x.

Castro-Cobo, S., Carpintero, S., Reyes-López, J.L., Sergio, F. and Angulo, E. (2019). Humans and scavenging raptors facilitate Argentine ant invasion in Doñana National Park: no counter-effect of biotic resistance. Biological Invasions 5. doi: https://doi.org/10.1007/s10530-019-01971-5.

Cole, F.R., Medeiros, A.C., Loope, L.L. and Zuehlke, W.W. (1992). Effects of the Argentine ant on arthropod fauna of the Hawai’ian high-elevation. Ecology 73(4):1313–1322.

Darracq, A.K., Smith, L.L., Oi, D.H., Conner, L.M. and McCleery, R.A. (2017). Invasive ants influence native lizard populations. Ecosphere 8(1). doi: https://doi.org/10.1002/ecs2.1657.

Davis, N.E., O’Dowd, D.J., Green, P.T. and Mac Nally, R. (2008). Effects of an alien ant invasion on abundance, behavior, and reproductive success of endemic island birds. Conservation Biology 22(5):1165–1176. doi: https://doi.org/10.1111/j.1523-1739.2008.00984.x.

Devenish, A.J.M., Newton, R.J., Bridle, J.R., Gomez, C., Midgley, J.J. and Sumner, S. (2021). Contrasting responses of native ant communities to invasion by an ant invader, Linepithema humile. Biological Invasions 23(8):2553–2571. doi: https://doi.org/10.1007/s10530-021-02522-7.

DiGirolamo, L.A. and Fox, L.R. (2006). The influence of abiotic factors and temporal variation on local invasion patterns of the Argentine ant (Linepithema humile). Biological Invasions 8(2):125–135. doi: https://doi.org/10.1007/s10530-004-1572-z.

Drescher, J., Feldhaar, H. and Blüthgen, N. (2011). Interspecific Aggression and Resource Monopolization of the Invasive Ant Anoplolepis gracilipes in Malaysian Borneo. Biotropica 43(1):93–99. doi: https://doi.org/10.1111/j.1744-7429.2010.00662.x.

Dunham, A.E. and Mikheyev, A.S. (2010). Influence of an invasive ant on grazing and detrital communities and nutrient fluxes in a tropical forest. Diversity and Distributions 16(1):33–42. doi: https://doi.org/10.1111/j.1472-4642.2009.00620.x.

Estany-Tigerström, D., Bas, J.M. and Pons, P. (2010). Does Argentine ant invasion affect prey availability for foliage-gleaning birds? Biological Invasions 12(4):827–839. doi: https://doi.org/10.1007/s10530-009-9504-6.

Gasc, A., Anso, J., Sueur, J., Jourdan, H. and Desutter-Grandcolas, L. (2018). Cricket calling communities as an indicator of the invasive ant Wasmannia auropunctata in an insular biodiversity hotspot. Biological Invasions 20(5):1099–1111. doi: https://doi.org/10.1007/s10530-017-1612-0.

Guénard, B. and Dunn, R.R. (2010). A new (Old), invasive ant in the hardwood forests of eastern North America and its potentially widespread impacts. PLoS ONE 5(7):1–10. doi: https://doi.org/10.1371/journal.pone.0011614.

Hanna, C., Naughton, I., Boser, C., Alarcón, R., Hung, K.L.J. and Holway, D. (2015). Floral visitation by the Argentine ant reduces bee visitation and plant seed set. Ecology 96(1):222–230. doi: https://doi.org/10.1890/14-0542.1.

Hoffmann, B.D., Andersen, A.N. and Hill, G.J.E. (1999). Impact of an introduced ant on native rain forest invertebrates: Pheidole megacephala in monsoonal Australia. Oecologia 120(4):595–604. doi: https://doi.org/10.1007/s004420050895.

Hoffmann, B.D., Auina, S. and Stanley, M.C. (2014). Targeted Research to Improve Invasive Species Management: Yellow Crazy Ant Anoplolepis gracilipes in Samoa. PLoS ONE 9(4):e95301. doi: https://doi.org/10.1371/journal.pone.0095301.

Hoffmann, B.D. and Parr, C.L. (2008). An invasion revisited: The African big-headed ant (Pheidole megacephala) in northern Australia. Biological Invasions 10(7):1171–1181. doi: https://doi.org/10.1007/s10530-007-9194-x.

Jourdan, H., Sadlier, R.A. and Bauer, A.M. (2001). Little Fire Ant Invasion (Wasmannia auropunctata) as a Threat to New Caledonian Lizards: Evidences from a Sclerophyll Forest (Hymenoptera: Formicidae). Sociobiology 38(3A):283–301.

Krushelnycky, P.D. and Gillespie, R.G. (2008). Compositional and functional stability of arthropod communities in the face of ant invasions. Ecological Applications 18(6):1547–1562. doi: https://doi.org/10.1890/07-1293.1.

Lach, L. (2007). A mutualism with a native membracid facilitates pollinator displacement by Argentine ants. Ecology 88(8):1994–2004. doi: https://doi.org/10.1890/06-1767.1.

Lach, L. (2008). Argentine ants displace floral arthropods in a biodiversity hotspot. Diversity and Distributions 14(2):281–290. doi: <https://doi.org/10.1111/j.1472-4642.2007.00410.x>.

Lach, L., Case, D., Yeeles, P. and Hoskin, C. J. (2022). Invasive ants reduce abundance of small rainforest skinks. Biodiversity and Conservation 31:739-755. https://doi.org/10.1007/s10531-022-02360-6

McPhee, K., Garnas, J., Drummond, F. and Groden, E. (2012). Homopterans and an invasive red ant, Myrmica rubra (L.), in Maine. Environmental Entomology 41(1):59–71. doi: https://doi.org/10.1603/EN11046.

Menke, S.B., Ward, P.S. and Holway, D.A. (2018). Long-term record of Argentine ant invasions reveals enduring ecological impacts. Ecology 99(5):1194–1202. doi: https://doi.org/10.1002/ecy.2200.

Milligan, P.D., Prior, K.M. and Palmer, T.M. (2016). An invasive ant reduces diversity but does not disrupt a key ecosystem function in an African savanna. Ecosphere 7(10):1–8. doi: https://doi.org/10.1002/ecs2.1502.

Morris, J.R. and Steigman, K.L. (2018). Southwestern Association of Naturalists Effects of Polygyne Fire Ant Invasion on Native Ants of a Blackland Prairie in Texas Author ( s ): John R . Morris and Kenneth L . Steigman Published by : Southwestern Association of Naturalists Stable URL : http://. 38(2):136–140.

Morrow, M.E., Chester, R.E., Lehnen, S.E., Drees, B.M. and Toepfer, J.E. (2015). Indirect effects of red imported fire ants on Attwater’s prairie-chicken brood survival. Journal of Wildlife Management 79(6):898–906. doi: https://doi.org/10.1002/jwmg.915.

Naughton, I., Boser, C., Tsutsui, N.D. and Holway, D.A. (2020). Direct evidence of native ant displacement by the Argentine ant in island ecosystems. Biological Invasions 22(2):681–691. doi: https://doi.org/10.1007/s10530-019-02121-7.

Naumann, K. and Higgins, R.J. (2014). The European fire ant (Hymenoptera: Formicidae) as an invasive species: Impact on local ant species and other epigaeic arthropods. Canadian Entomologist 147(5):592–601. doi: https://doi.org/10.4039/tce.2014.69.

O’Dowd, D.J., Green, P.T. and Lake, P.S.P. (2003). Invasional ‘meltdown’ on an oceanic island. Ecology Letters 6(9):812–817. doi: https://doi.org/10.1046/j.1461-0248.2003.00512.x.

Ogura-Yamada, C.S. and Krushelnycky, P.D. (2020). The effects of the invasive thief ant, Solenopsis papuana, on ground-dwelling invertebrates in mesic forests of Hawaiʻi. Journal of Insect Conservation 24(1):151–162. doi: https://doi.org/10.1007/s10841-019-00185-3.

Porter, S.D. and Savignano, D.A. (1990). Invasion of polygyne fire ants decimates native ants and disrupts arthropod community. Ecology. doi: https://doi.org/10.2307/1938623.

Rowles, A.D. and O’Dowd, D.J. (2009). Impacts of the invasive Argentine ant on native ants and other invertebrates in coastal scrub in south-eastern Australia. Austral Ecology 34(3):239–248. doi: https://doi.org/10.1111/j.1442-9993.2008.01922.x.

Rowles, A.D. and Silverman, J. (2010). Argentine ant invasion associated with loblolly pines in the South eastern United States: Minimal impacts but seasonally sustained. Environmental Entomology 39(4):1141–1150. doi: https://doi.org/10.1603/EN10004.

Sahli, H.F., Krushelnycky, P.D., Drake, D.R. and Taylor, A.D. (2016). Patterns of Floral Visitation to Native Hawaiian Plants in Presence and Absence of Invasive Argentine Ants. Pacific Science 70(3):309–322. doi: https://doi.org/10.2984/70.3.3.

Salyer, A., Bennett, G.W. and Buczkowski, G.A. (2014). Odorous house ants (Tapinoma sessile) as back-seat drivers of localized ant decline in urban habitats. PLoS ONE 9(12):1–14. doi: https://doi.org/10.1371/journal.pone.0113878.

Sarty, M., Abbott, K.L. and Lester, P.J. (2007). Community level impacts of an ant invader and food mediated coexistence. Insectes Sociaux 54(2):166–173. doi: https://doi.org/10.1007/s00040-007-0927-8.

Stork, N.E., Kitching, R.L., Davis, N.E. and Abbott, K.L. (2014). The impact of aerial baiting for control of the yellow crazy ant, Anoplolepis gracilipes, on canopy-dwelling arthropods and selected vertebrates on Christmas Island (Indian Ocean). Raffles Bulletin of Zoology 7600(30):81–92.

Verble-Pearson, R. and Pearson, S. (2016). European fire ant presence decreases native arboreal insect abundance in Acadia National Park, Maine, USA. Natural Areas Journal 36(2):162–165. doi: https://doi.org/10.3375/043.036.0207.

Walker, K.L. (2006). Impact of the little fire ant, Wasmannia auropunctata, on native forest ants in Gabon. Biotropica. doi: https://doi.org/10.1111/j.1744-7429.2006.00198.x.

Wong, M.K.L., Guénard, B. and Lewis, O.T. (2020). The cryptic impacts of invasion: functional homogenization of tropical ant communities by invasive fire ants. Oikos 129(4):585–597. doi: https://doi.org/10.1111/oik.06870.
